# An exploratory study of breathwork-induced altered states of consciousness in experienced practitioners: the airways to alteration (A2A) trial

**DOI:** 10.3389/fpsyg.2026.1851882

**Published:** 2026-06-10

**Authors:** Guy W. Fincham, Edward Caddye, Amy A. Kartar, Elizabeth A. Lilley, Nicola Stoke, Alessandro Colasanti

**Affiliations:** 1 Breathwork Lab, Department of Clinical Neuroscience, Brighton & Sussex Medical School, Brighton and Hove, United Kingdom; 2 Psychoactive Trials Group, Institute of Psychiatry, Psychology & Neuroscience, King’s College London, London, United Kingdom; 3 Independent Scholar, London, United Kingdom; 4 Sussex Partnership NHS Foundation Trust, Worthing, United Kingdom

**Keywords:** altered states of consciousness, behavior change, breathwork, emotional breakthrough, high ventilation, meditation, mystical experience, psychological insight

## Abstract

**Background:**

Breathwork that increases ventilatory rate or depth represents an accessible non-pharmacological modality for potentially inducing altered states of consciousness (ASCs). Despite gaining traction as a potential therapeutic tool, empirical controlled research on breathwork and ASCs remains limited.

**Methods:**

We examined the effects of a single session of high ventilation breathwork, compared to body scan meditation, in 24 healthy adults with primary outcomes of acute ASCs including mystical experience and emotional breakthrough. Sub-acute secondary outcomes were collected 1 week later.

**Results:**

Breathwork was associated with larger effects on oceanic boundlessness (*p* = 0.007, *r* = 0.63), visionary restructuralisation (*p* = 0.018, *r* = 0.60), total mystical experience (*p* = 0.007, *r* = 0.66), oneness (*p* = 0.018, *r* = 0.60), positive mood (*p* = 0.007, *r* = 0.66), ineffability (*p* = 0.038, *r* = 0.55), and emotional breakthrough (*p* = 0.028, *r* = 0.45). At follow-up, breathwork was associated with substantially greater psychological insight (*p* = 0.002, *r* = 0.67) and behavioral change (*p* = 0.008, *r* = 0.60) relative to body scan meditation. Stress, anxiety, depression and wellbeing improved in both groups over time.

**Discussion:**

Results from this preliminary experimental study indicate that breathwork is associated with larger acute psychedelic-like effects than meditation, alongside greater emotional breakthrough, insight, and self-reported behavioral change. These exploratory relationships and preliminary observations provide greater context around breathwork-induced ASCs, and support the feasibility of ASC-focused breathwork research for future confirmatory trials.

## Introduction

Breathing, rooted in the Latin word for breath “*spiritus*,” i.e., spirit, is fundamental to life. Beyond its essential physiological function (Del Negro et al., 2018), breathing has served as one of the most accessible gateways to altered states of consciousness (ASCs) across cultures and throughout history (Fincham et al., 2023a), showcasing great anthropological value. High ventilation breathwork (HVB), defined as mind-body practices that deliberately increase respiratory rate and/or depth, has been used to relieve various forms of psychological distress for millennia (Fincham et al., 2023a). The most commonly recognized manifestation of breathwork is found in Pranayama, one of the classical limbs of Yoga, which concerns the regulation of vital energy (*prana* = life force; *ayama* = control). Most breathwork research to date has focused on slow-paced techniques (Fincham et al., 2023c,d), however the field of HVB research in particular remains in its “embryonic” stage.

Breathing remains a central focus of several contemplative traditions, with the ultimate aim of reducing suffering. Both ancient slow and fast-paced breath techniques have been practised in yogic traditions, including Nadi Shodhana (alternate nostril breathing) and Bhastrika (bellows breath) (Iyengar, 1981), with more recent modalities emerging such as Sudarshan Kriya (Korkmaz et al., 2024). Additionally, breath practices are traditionally and practically significant to other biofield healing systems like Qigong, where *qi* may be considered synonymous with *prana* (Rubik et al., 2015; Belal et al., 2023). Within Qigong, breath is used not only to cultivate internal *qi* but to direct it therapeutically, both within the practitioner’s own body and, in advanced practice, toward others (Jahnke et al., 2010). Beyond yogic and Qigong lineages, intentional breath practices play a role in traditions worldwide including Sufi *dhikr* (Frager, 2005) and shamanic practices (Winkelman, 2010), suggesting that the therapeutic use of breath represents one of humanity’s most consistent cross-cultural healing technologies.

Psychedelic substances represent the most well-documented pathway to ASCs and have therapeutic potential, however they face significant medical, legal and financial barriers (Havenith et al., 2025). Breathwork is not restricted in the same way and offers an immediate, drug-free alternative. Recent research has shown that HVB can produce profound alterations in consciousness (Lewis-Healey et al., 2024; Havenith et al., 2025). In a structured context, where attention is paid to the set and setting, HVB has been reported to produce large effects on validated ASC measures (e.g., MEQ-30, 5D-ASC), with magnitudes of effect falling within the range observed in studies administering moderate-to-high doses of psilocybin (Bahi et al., 2024). Clinical observations and preliminary neurophysiological studies indicate that HVB is associated with changes in subjective experience and intense effects on central and autonomic nervous system functions through modulation of neurometabolic parameters and interoceptive sensory systems (Fincham et al., 2023a; Kartar and Colasanti, 2025). A recent study revealed that the intensity of ASCs induced by breathwork was proportional to cerebral hemodynamic changes (cerebral blood flow; CBF) measured by quantitative magnetic resonance imaging (MRI) and to the degree of autonomic nervous system perturbations (heart rate variability; HRV) (Kartar et al., 2025).

Controlled studies have demonstrated that breathwork practices lower levels of stress and self-reported anxiety and depressive symptoms (Fincham et al., 2023d). HVB, in particular, may prove beneficial for conditions such as post-traumatic stress disorder (PTSD) (Bayley et al., 2022). However, no study to our knowledge has examined the effects of breathwork on ASCs through a randomized-controlled design, representing a critical gap in our understanding of this emerging modality.

Specifically, two prior studies by Bahi et al. (2024) and Havenith et al. (2025) provided a preliminary evaluation of HVB-induced ASCs, but these studies lacked a control group. The former was also conducted in a mixed group of participants with and without prior experience of HVB. The effects of expectancy and prior experience, which play a role in all contemplative practices like mindfulness meditation (Wielgosz et al., 2019), are also likely to mediate, at least in part, the effects of HVB on ASCs. Moreover, previous studies did not determine whether the psychological benefits were short-lived effects that depended on the acute ASCs during the breathing sessions, or whether they continued after the sessions ended. Assessing whether the psychological impact persists beyond the acute experience is a question of great relevance to characterize the putative mechanisms of HVB practices and their potential in clinical applications (Fincham et al., 2024). The present study addresses these gaps by adopting a randomized controlled design to examine the effects of breathwork on ASCs.

Our choice of study design and methods were aimed to specifically address those outstanding questions: first, we focused on conscious connected breathing (CCB), one of the most popular and accessible forms of HVB, which comprises intensive circular breathing (without pauses between in and out breath) paired with music. CCB has been reported to induce ASCs (Bahi et al., 2024), to an extent comparable to those induced by longer Holotropic Breathwork sessions (Havenith et al., 2025).

Second, we compared the effects of CCB to those of a body scan meditation in a controlled experimental design. Body scan meditation is frequently taught as a foundational practice in mindfulness-based programmes (Williams and Penman, 2011), and like CCB, is delivered in a supine position (Kabat-Zinn, 2016). It allowed us to control for the set and setting (including the space) in which it was delivered. Like the practice of Yoga Nidra (Pandi-Perumal et al., 2022), it involves lying down and passing attention through different parts of the body (Fincham et al., 2023b). Importantly, we considered that the selection of an established contemplative technique such as meditation might provide an active comparator since mindfulness and other forms of meditation have been associated with ASCs (Galante et al., 2024) including mystical experiences (Woollacott et al., 2024).

Third, we examined the acute effects experienced immediately after the session. We specifically chose scales that are frequently administered in the psychedelic literature, and some used in prior breathwork research, which could potentially capture breathwork-induced altered states relating to emotional breakthrough (Roseman et al., 2019), mystical experience (Barrett et al., 2015; Strickland et al., 2024), and other ASCs like oceanic boundlessness. In particular, the latter encompasses a cluster of related experiences including spiritual states, feelings of insight, blissfulness, positively valenced depersonalization, and a sense of unity with the world (Studerus et al., 2010). Furthermore, we included a more specific measure of depersonalization (Schweden et al., 2019) since hyperventilation may cause or exacerbate such feelings (Lickel et al., 2008). Additionally, we included sub-acute insight, health and wellbeing-related outcomes persisting 1 week after the meditation and breathwork sessions. This provided enough time for participants to go about their daily lives over a weekly cycle, potentially integrate any changes and observe them, but without too long of a follow-up period.

Last, we focused our study sample on experienced breathwork practitioners to minimize inter-individual heterogeneity in the degree of expectancy and experience and their impact on psychological outcomes. Therefore, we conducted a proof-of-concept randomized controlled study to provide a preliminary examination of the phenomenological effects of a single session of CCB versus a body scan meditation. The primary objective was to estimate acute ASC outcomes of putative clinical relevance, such as mystical experiences and emotional breakthrough, and to generate effect size estimates to inform the design of future, adequately powered trials.

## Materials and methods

### Randomization and blinding

This study employed a proof-of-concept, parallel-group, randomized controlled design with an open-label allocation. Participants were informed that they would be randomly assigned to either a CCB or body scan meditation session but were unaware of their allocation until arrival on the study day. Randomization was conducted using block randomization (1:1), stratified by scores on the Imperial Psychedelic Predictor Scale (IPPS), which was completed shortly after informed consent. Three cohabiting participant pairs (six individuals) were allocated to the same condition to preserve procedural fidelity, consistent with prior breathwork RCT designs (Balban et al., 2023). Randomization was performed by a researcher external to the study team who had access only to participant IDs and IPPS scores.

### Participants

A total of 24 participants were recruited, with 12 allocated to each condition. Previous reports have suggested large effects of CCB on ASC measures (Bahi et al., 2024; Havenith et al., 2025), however since our study had an exploratory, hypothesis-generating character, this meant a pilot sample size justification was more appropriate than a confirmatory framing. Consistent with the proof-of-concept nature of the study, a sample size of 12 participants per group was considered appropriate from a feasibility perspective (six participants per breathwork facilitator) and sufficient to provide preliminary estimates of effect magnitude and variability (rather than definitive efficacy conclusions) to potentially guide the design of future studies.

Inclusion criteria: relatively healthy adults 18–65 years old, residing in Brighton and surrounding area, having practiced CCB at least once before with no history of adverse events during such breathwork.

Exclusion criteria: history of: hypotension or hypertension; respiratory or cardiovascular conditions; fainting or syncope; epilepsy or seizures; panic disorder or panic attacks; cerebral aneurysm; breathlessness, bradypnea or tachypnea; any other mental/physical issues affecting the ability to engage in breath control activities. Lastly, pregnancy (and possibility one might be pregnant, trying to get pregnant, or are breastfeeding).

### Procedure

Participants were recruited using online advertisements (social media/email). Interested individuals were required to contact the principal investigator. Prospective participants were then screened in line with the eligibility criteria. Both meditation and breathwork sessions took place within a naturalistic setting where the main facilitator has regularly taught HVB in the form of CCB. Both breathwork and meditation were led by the same facilitator, with a co-facilitator present to provide support if needed. Both facilitators had also facilitated body scan meditation. All questionnaires were administered via the online platform Qualtrics^1^ with participants receiving phased monetary compensation per questionnaire completed to maximize retention. Additionally, all participants received their assigned intervention (breathwork or meditation) free of charge. The sessions happened on the same day (June 8, 2025) with breathwork in the afternoon, and meditation in the morning.

### Comparator

The active comparison condition consisted of a guided body scan meditation session. Participants were provided with mats, pillows and blankets for comfort in the designated room. The session began with a comprehensive orientation that included: welcome and space introduction, session outline, meditation instruction, confidentiality protocols, consent procedures (regarding touch) and integration discussion. Participants were invited to share their names and current feelings with the group. Following the initial orientation, participants engaged in spatial awareness exercises, observing the room’s colors, textures, edges, windows and doors while assessing their personal safety and comfort levels. Any additional comfort needs were addressed at this time. The group then participated in a brief somatic exercise called “Centering,” derived from the Generative Somatics methodology developed by Staci Haines^2^.

Participants subsequently positioned themselves laying on their mats and were provided with eye masks to aid self-reflection. Soft background music was selected from portions of the breathwork playlist and played at low volume throughout the session. The meditation protocol began with several minutes of coherent breathing (Fincham et al., 2023c) at six breaths per minute, followed by gentle, restful breathing during the continuously guided 40-min body scan meditation. The 40-min duration was specifically chosen to prevent participants from falling asleep, as this practice can be highly relaxing. Moreover, this time length is in line with Mindfulness-Based Stress Reduction (MBSR) which uses the scan as its first, foundational practice (Santorelli et al., 2017). Post-meditation grounding was facilitated through the provision of food (dates and vegan chocolate). The session concluded with an optional sharing circle within which participants could discuss their experiences prior to formal space closure.

### Intervention

The intervention involved a HVB session utilizing a CCB style of breathwork. This method employed continuous mouth breathing with active inhalation, passive exhalation, and no breath retention or pauses. Initial participant preparation mirrored the meditation session regarding consent, confidentiality and integration protocols. The session introduction included facilitator discussion of potentially expanded consciousness states, guided introspection for participants to identify any personal intentions, along with instruction and practice of the CCB technique. Participants were informed that the primary breathing pattern involved mouth breathing throughout the session, with the option to transition to nasal CCB if they desired to soften their experience.

Facilitators provided comprehensive preparation regarding potential session phenomena, including body temperature fluctuation, tetany and emotional expression (not provided in the comparator group in order to maintain ecological validity of the intended body scan meditation). All forms of emotional expression were explicitly permitted and participants were encouraged to request facilitator support as needed. No physical support was requested during the study. Participants were informed that the musical accompaniment was intentionally designed to follow the session’s trajectory, beginning with loud, fast-paced music and gradually slowing over the session duration. Eye masks were provided and earplugs were also made available for participants requiring them. Following name sharing and the same spatial orientation and somatic exercise used in the comparator condition, participants positioned themselves on mats with additional cushions and blankets available for comfort. The session commenced with several minutes of coherent nasal breathing before transitioning to mouth-based CCB. The music progression followed the predetermined loud-to-soft pattern and participants were invited to return to nasal breathing at the 70-min mark if they had not already done so (total session duration was 90 min). The session conclusion was identical to the comparator.

### Outcome measures

**FIGURE 1:**
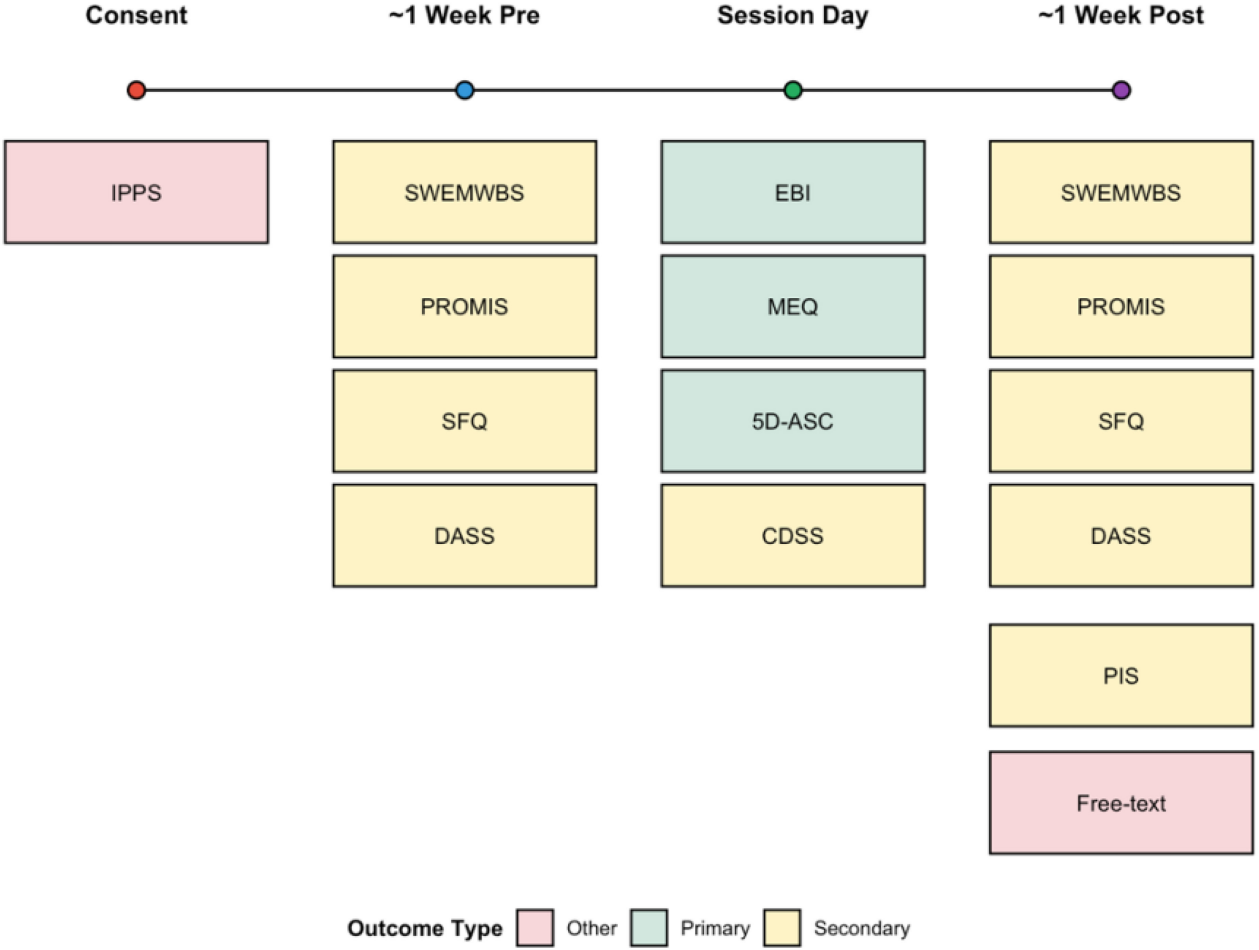
Timeline of outcome measures collected across the study. IPPS, Imperial Psychedelic Predictor Scale; SWEMWBS, Short Warwick-Edinburgh Mental Wellbeing Scale; PROMIS, PROMIS Item Bank v. 1.0 - Sleep-Related Impairment - Short Form 4a; SFQ, Shortened Fatigue Questionnaire; DASS, Depression Anxiety Stress Scale-21; EBI, Emotional Breakthrough Inventory; MEQ, Mystical Experience Questionnaire Brief; 5D-ASC, Five Dimensions of Altered States of Consciousness Scale; CDSS, Cambridge Depersonalization Scale; PIS, Psychological Insight Scale.

Figure 1 shows a timeline of all the outcome measures (primary, secondary, other) collected across the study (consent stage, baseline, day of session, follow-up).

One scale was completed immediately after obtaining informed consent (see Figure 1): Imperial Psychedelic Predictor Scale (IPPS) (Angyus et al., 2024). Comprises nine items, with scores for each item ranging from 0 to 100. Higher scores denote higher predictiveness of having a psychedelic experience. McDonald’s Omega (ω) demonstrated good internal consistency (9 items; ω = 0.88).

### Baseline and follow-up

Four scales were administered ∼1 week pre-post intervention (Figure 1):

Shortened Fatigue Questionnaire (SFQ) (Penson et al., 2020). Comprises four items. Scores range from 4 to 28, with higher scores indicating more severe fatigue. McDonald’s Omega demonstrated good internal consistency at baseline and post-intervention (4 items; ω = 0.83 and 0.86, respectively).

PROMIS Item Bank v. 1.0 - Sleep-Related Impairment - Short Form 4a (PROMIS) (Hanish et al., 2017). Comprises four items. Scores range from 5 to 20, with higher scores denoting higher levels of sleep-related impairment. Data are scored using a T-score transformation according to the PROMIS Sleep scoring manual^3^. McDonald’s Omega demonstrated excellent internal consistency at baseline and post-intervention (4 items; ω = 0.92 and 0.97, respectively).

Short Warwick-Edinburgh Mental Wellbeing Scale (SWEMWBS) (Ng Fat et al., 2017). Comprises seven items. Scores range from 7 to 35, with higher scores denoting greater wellbeing. Total raw scores are then transformed into metric scores using the SWEMWBS conversion table^4^. McDonald’s Omega demonstrated good internal consistency at baseline and post-intervention (7 items; ω = 0.81 and 0.84, respectively).

Depression Anxiety Stress Scale-21 (DASS) (Lovibond and Lovibond, 1995). Comprises 21 items and three subscales: stress, anxiety and depression, with scores ranging from 0 to 21 for each. Higher scores denote worse outcomes. Scores are then multiplied by two to convert it to the longer form DASS-42 final score. McDonald’s Omega demonstrated good internal consistency at baseline and post-intervention (21 items; ω = 0.83 and 0.84, respectively).

### Immediate post-session

The primary outcomes of potential altered states of consciousness (ASCs) emerging from the meditation and breathwork were measured using three scales immediately after the sessions (Figure 1):

Mystical Experience Questionnaire Brief (MEQ) (Strickland et al., 2024). Comprises four items with a corresponding subscale for each: transcendence (of time and space), positive mood, ineffability (i.e., incapable of being expressed or described in words) and mystical or oneness (sense of oneness, insight into ultimate reality, or sacredness). Scores range from 0 to 20, with higher scores indicating a more profound mystical experience. McDonald’s Omega demonstrated good internal consistency (4 items; ω = 0.88).

Emotional Breakthrough Inventory (EBI) (Roseman et al., 2019). Comprises six items. Scores range from 0 to 100 (total of 600), with higher scores indicating greater levels of emotional breakthrough. McDonald’s Omega demonstrated excellent internal consistency (6 items; ω = 0.94).

Five Dimensions of Altered States of Consciousness Scale (5D-ASC) (Dittrich, 1998). Comprises 94 items and five main scales: oceanic boundlessness, dread of ego dissolution, visionary restructuralisation, auditory alterations and vigilance reduction. Scores range from 0 to 100, with higher scores indicating more profound ASCs. McDonald’s Omega demonstrated excellent internal consistency (94 items; ω = 0.96).

Another scale was completed immediately after the sessions (Figure 1): Cambridge Depersonalization Scale (State-Version; CDSS) (Schweden et al., 2019). Comprises 22 items. Scores range from 0 to 100 (total of 2,200), with higher scores indicating higher levels of dissociative symptoms. McDonald’s Omega demonstrated good internal consistency (22 items; ω = 0.88).

### Follow-up only

Around 1 week after the sessions, another scale was completed (Figure 1): Psychological Insight Scale (PIS) (Peill et al., 2022). Comprises seven items (including one additional–see below). Scores range from 0 to 100, with higher scores indicating greater psychological insight. An additional item separately evaluates self-reported behavioral change resulting from a psychedelic experience. McDonald’s Omega demonstrated excellent internal consistency (7 items; ω = 0.96).

Participants were also able to share their general experiences 1-week post-intervention through open-ended free text. The item read: “Do you have anything you would like to add about your experience during the breathwork or meditation session, and/or overall study period?” This short amount of text data were intended to simply complement the primary and secondary outcomes.

### Data analysis

Data analyses were conducted using the software R (R-Core-Team, 2021). Outcome measures collected immediately after the meditation and breathwork session were analyzed using Wilcoxon rank-sum tests (McKnight and Najab, 2010) as normality was violated for many variables based on Shapiro-Wilk tests (González-Estrada and Cosmes, 2019). This was likely due to the small sample sizes per group; in line with the central limit theorem, *n* ≥ 30 is often cited as a rule of thumb for assuming normality (Kwak and Kim, 2017). Baseline and follow-up (around 1 week pre-post breathwork/meditation) outcomes were analyzed with non-parametric factorial analyses using Aligned Rank Transform (ART) (Wobbrock et al., 2011) (only significant group × time interactions followed up), again due to several normality violations and the small sample size. Psychological insight and accompanied behavioral change were analyzed using Wilcoxon rank-sum test, since this was collected only once, ∼1 week after the sessions, and was not a repeated-measure. Accordingly, medians (Mdn) and interquartile ranges (IQR) were reported throughout owing to the nature of these non-parametric tests. Spearman rank-order correlations (Ramsey, 1989) were also used to assess the correlation between mental health/wellbeing changes and ASC measures, along with IPPS scores and ASCs. Where subscales were present, Bonferroni corrections (Haynes, 2013) were applied to control for multiple comparisons. Simple sentiment analysis was used for the short amount of free text data on general experience.

### Ethics approval

Ethical approval was obtained from the Brighton and Sussex Medical School Research Governance and Ethics Committee (ERA/GF221/9/1). The study was preregistered with ClinicalTrials.gov (NCT06916312) and conducted in accordance with the tenets of the Declaration of Helsinki. All participants provided informed consent.

## Results

### Baseline

Study recruitment began February 26 and was completed May 14, 2025. In line with Supplementary Figure 1 (CONSORT flow diagram), 24 participants were recruited and all completed both the primary and secondary outcome measures. Table 1 displays the baseline demographics and measure scores–there were no significant differences across these.

**TABLE 1:** Baseline demographics and measures.

Characteristic	Group		
Characteristic	Breathwork (*n* = 12)	Meditation (*n* = 12)	Test statistic (*p*)
Gender (*n*)			*X*^2^_2_ ≅ 0.21 (0.90)
Woman	8	7	
Man	3	4	
Non-binary	1	1	
Age [Mdn (IQR)]	45 (30.5–52.2)	43 (33–45)	*W* = 88 (0.37)
IPPS	76.6 (71.2–80.3)	75.8 (69.5–81.8)	*W* = 75 (0.89)
DASS	22 (17.5–34.5)	27 (19.5–36)	*W* = 62 (0.58)
Stress	12 (10–16)	12 (11–14)	*W* = 75.5 (0.86)
Anxiety	5 (2–8)	6 (2–12)	*W* = 59 (0.46)
Depression	4 (0–13)	10 (3.5–12)	*W* = 54.5 (0.32)
SWEMWBS	23.2 (21.2–25.5)	23.2 (20.5–24.3)	*W* = 77.5 (0.77)
PROMIS	54.8 (49–60.9)	54.3 (52.5–60.9)	*W* = 65 (0.71)
SFQ	16.5 (13.2–19.2)	13.5 (13–14.2)	*W* = 93.5 (0.22)

Mdn, median; IQR, interquartile range.

### Immediate post-session

#### Mystical experience

**FIGURE 2:**
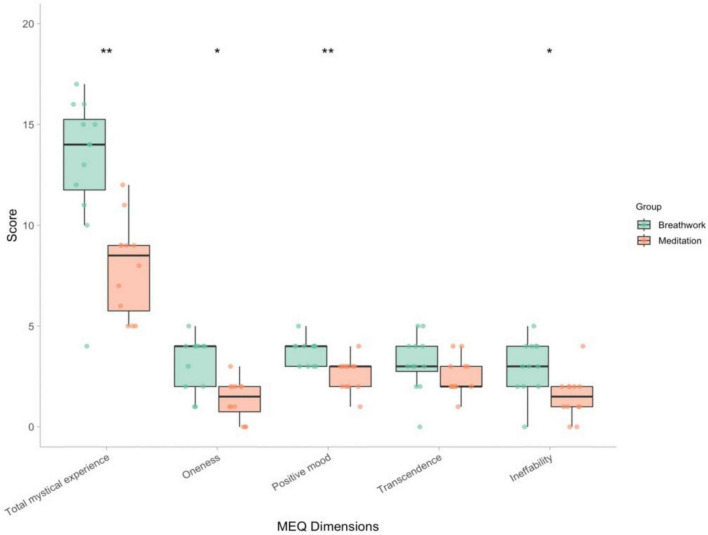
Boxplots of MEQ dimension scores by group (breathwork vs. meditation). Each box represents the interquartile range (IQR) with horizontal line indicating median score, and individual data points overlaid with jitter for visibility. Asterisks denote group differences based on Bonferroni-corrected Wilcoxon rank-sum tests (*p < 0.05  **p < 0.01).

Scores on the MEQ subscales and total scores differed between groups (see Figure 2). The breathwork group reported a higher total mystical experience score (Mdn = 14, IQR = 11.8–15.2) than the meditation group (Mdn = 8.50, IQR = 5.75–9). Wilcoxon rank-sum tests revealed significant group differences in total mystical experience (*W* = 128, *p* = 0.001, Bonferroni-adjusted *p* = 0.007, large effect size, *r* = 0.66), oneness/mystical (*W* = 122, *p* = 0.0037, adjusted *p* = 0.018, large effect size, *r* = 0.60), positive mood (*W* = 125, *p* = 0.0014, adjusted *p* = 0.007, large effect size, *r* = 0.66), and ineffability (*W* = 118, *p* = 0.008, adjusted *p* = 0.038, large effect size, *r* = 0.55). There was no significant difference in transcendence. Spearman rank-order (ρ) correlations revealed no significant associations between Imperial Psychedelic Predictor Scale (IPPS) scores and any MEQ dimension before or after Bonferroni correction.

#### Emotional breakthrough

**FIGURE 3:**
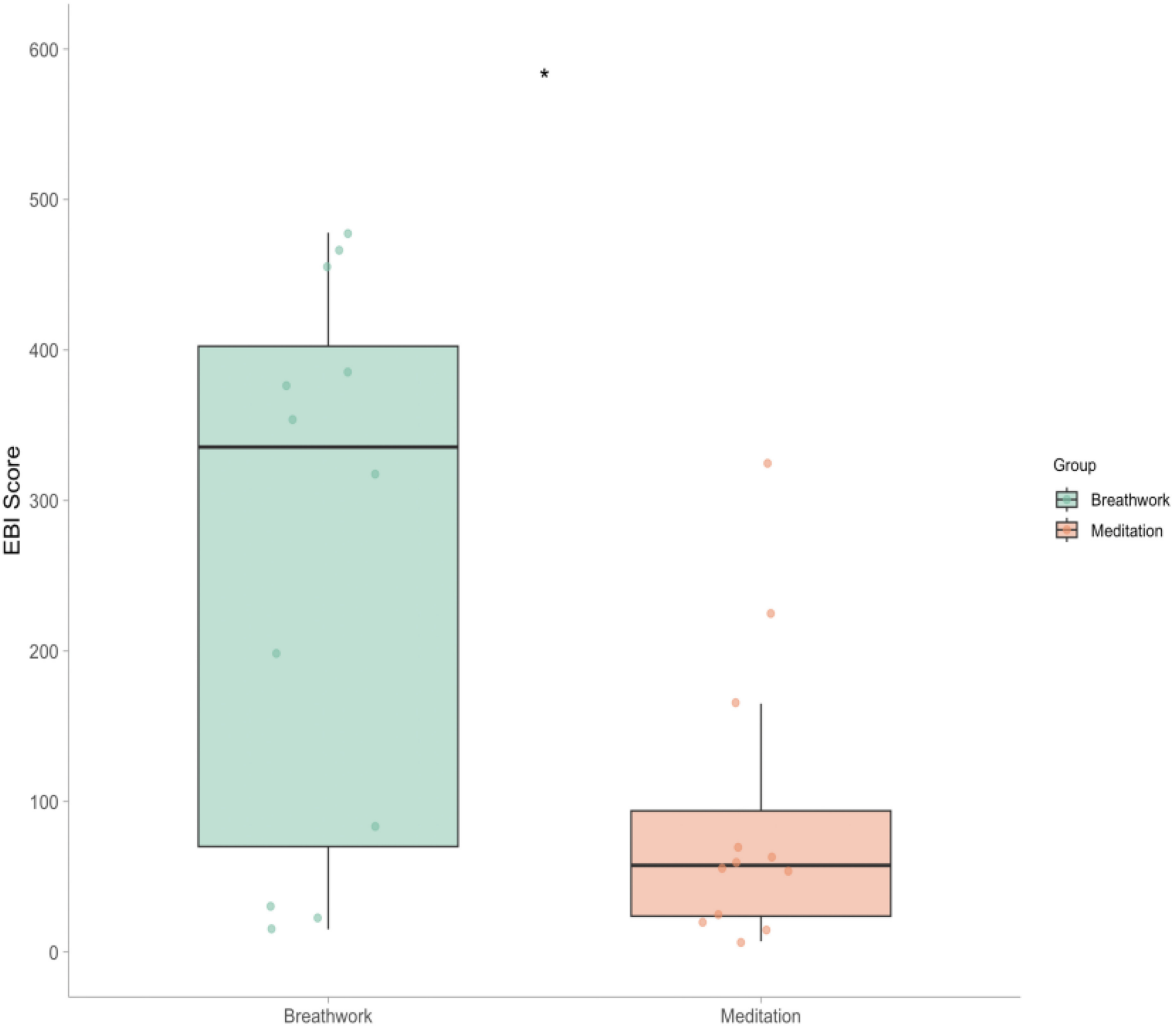
Boxplots of EBI scores by group (breathwork vs. meditation). Each box represents the interquartile range (IQR) with horizontal line indicating median score, and individual data points overlaid with jitter for visibility.

Emotional Breakthrough Inventory scores differed between groups (Figure 3). The breathwork group reported a significantly higher EBI score (Mdn = 336, IQR = 70.0–402) than the meditation group (Mdn = 57.5, IQR = 23.8–93.8), *W* = 110, *p* = 0.028, moderate effect size, *r* = 0.45. There was no significant correlation between IPPS and EBI scores.

#### Altered states of consciousness

**FIGURE 4:**
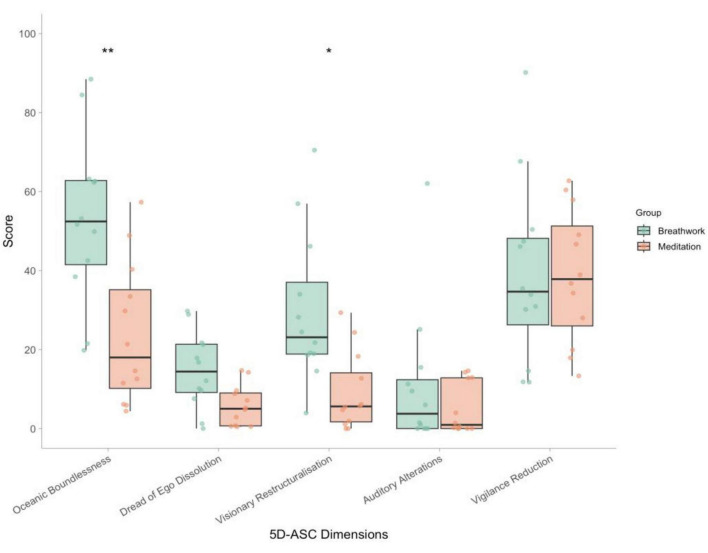
Boxplots of 5D-ASC dimension scores by group (breathwork vs. meditation). Each box represents the interquartile range (IQR) with horizontal line indicating median score, and individual data points overlaid with jitter for visibility. Asterisks denote group differences based on Bonferroni-corrected Wilcoxon rank-sum tests (*p < 0.05, **p < 0.01).

Median scores on the 5D-ASC differed between groups (Figure 4). The breathwork group reported numerically higher oceanic boundlessness (Mdn = 52.44, IQR = 41.50–62.80), dread of ego dissolution (Mdn = 14.43, IQR = 9.14–21.36), visionary restructuralisation (Mdn = 23.11, IQR = 18.86–37.04), auditory alterations (Mdn = 3.75, IQR = 0.00–12.36), and vigilance reduction (Mdn = 34.67, IQR = 26.27–48.17) compared to the meditation group (Mdn = 18.00, IQR = 10.18–35.16; Mdn = 5.02, IQR = 0.68–9.01; Mdn = 5.61, IQR = 1.69–14.11; Mdn = 0.94, IQR = 0.00–12.84; Mdn = 37.83, IQR = 25.98–51.29, respectively).

Wilcoxon rank-sum tests revealed significant group differences in oceanic boundlessness (*W* = 125, *p* = 0.001, Bonferroni-adjusted *p* = 0.007, large effect size, *r* = 0.63) and visionary restructuralisation (*W* = 123, *p* = 0.004, adjusted *p* = 0.018, large effect size, *r* = 0.60). The difference in dread of ego dissolution was not statistically significant after correction, and no significant group differences were observed for auditory alterations or vigilance reduction. There were no significant correlations between IPPS scores and all 5D-ASC dimensions before and after correction.

#### Depersonalization

There was no significant difference on CDSS scores between the breathwork group (Mdn = 184.50, IQR = 104.75–270.25) and meditation group (Mdn = 78, IQR = 64.25–148.00).

### Follow-up

#### Psychological insight and behavioral change

PIS scores differed substantially between groups. The breathwork group reported higher psychological insight (Mdn = 33.67, IQR = 19.96–50.58) and accompanied behavioral change (Mdn = 50, IQR = 20.50–61) than the meditation group (Mdn = 5.83, IQR = 1.88–7.54; Mdn = 5.50, IQR = 4.50–7.25, respectively). Wilcoxon rank-sum tests showed significant group differences in both psychological insight (*W* = 129, *p* = 0.001, Bonferroni-adjusted *p* = 0.002, large effect size, *r* = 0.67) and behavioral change (*W* = 122, *p* = 0.004, adjusted *p* = 0.008, large effect size, *r* = 0.60).

#### Stress, anxiety and depression

**FIGURE 5:**
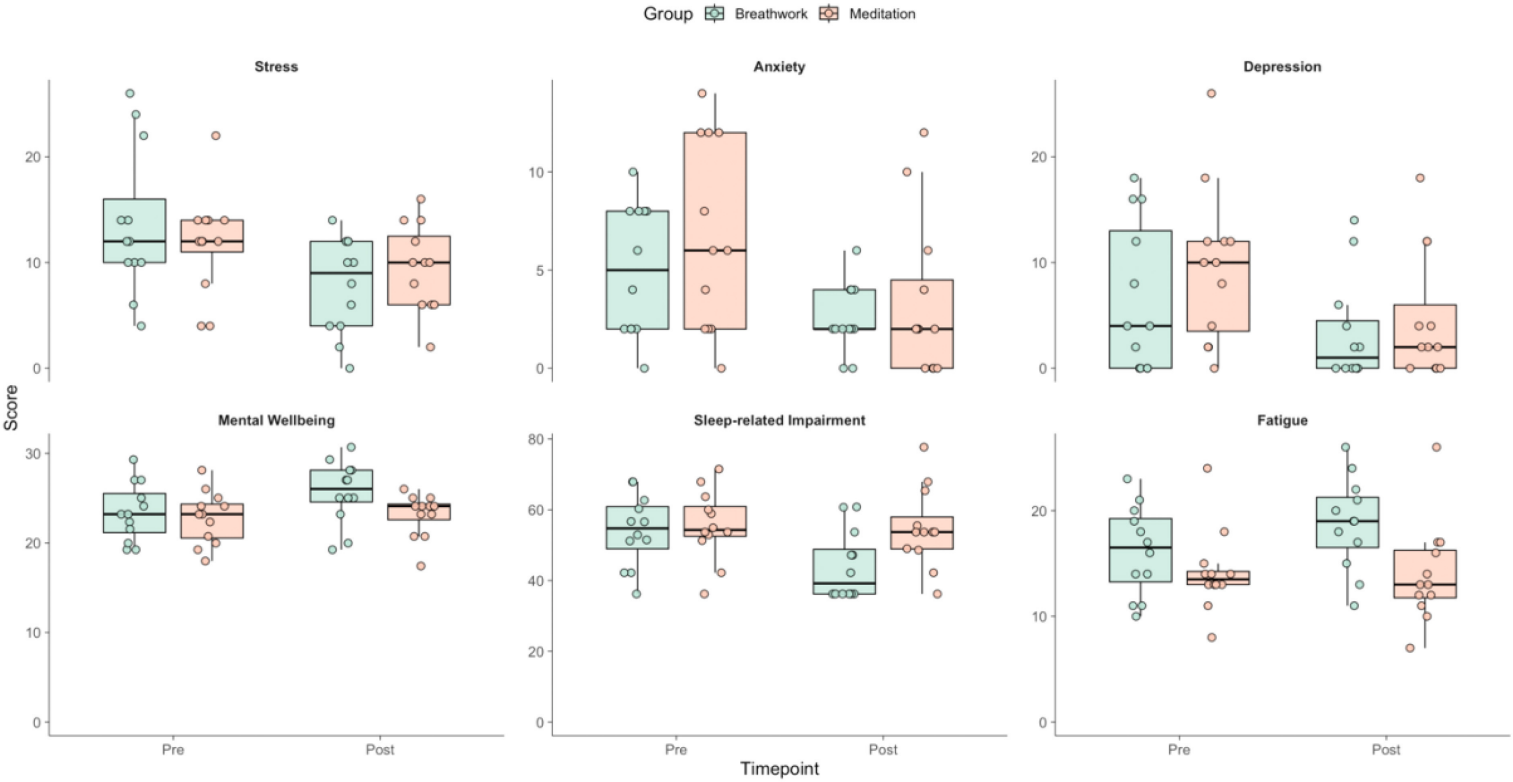
Boxplots of psychological outcome measures before and after intervention (pre vs. post) by group (breathwork vs. meditation). Each panel represents one outcome variable: stress, anxiety, depression (DASS), mental wellbeing (SWEMWBS), sleep-related impairment (PROMIS), and fatigue (SFQ). Boxes represent interquartile range (IQR) with horizontal lines indicating the median. Individual data points are overlaid with jitter for visibility. Scores are plotted separately for breathwork (green) and meditation (orange) groups. *Y*-axis scales are adjusted per variable, but all include zero for reference.

Non-parametric factorial analyses using Aligned Rank Transform (ART) revealed significant main effects of time for all three DASS subscale outcomes – stress, anxiety and depression – indicating reductions from pre- to post-intervention across groups (Figure 5). Bonferroni-corrected *p*-values confirmed that these effects remained statistically significant (adjusted *p* = 0.0069, 0.0007, and 0.0027, respectively). However, no significant group × time interactions were found for any of the subscales, indicating that the magnitude of change did not differ significantly between the breathwork and meditation groups. No significant correlations were found between primary outcomes EBI and 5D-ASC and differences on pre-post DASS scores; however, a significant negative correlation was found only for the MEQ subscale of positive mood and stress (ρ = −0.64, *p* < 0.001, Bonferroni-adjusted *p* = 0.004).

#### Mental wellbeing

Non-parametric factorial analysis using ART revealed a significant main effect of time, *F*(1, 22) = 4.61, *p* = 0.043, suggesting that SWEMWBS scores increased from pre- to post-intervention (Figure 5). However, there was no significant group × time interaction, indicating that the amount of change on mental wellbeing did not differ significantly between groups. No significant correlations were found between the primary outcomes and changes in mental wellbeing.

#### Sleep-related impairment

Non-parametric factorial analysis revealed a significant main effect of time, *F*(1, 22) = 10.1, *p* = 0.004, and a significant group × time interaction, *F*(1, 22) = 8.68, *p* = 0.007, indicating that the decrease in PROMIS scores from pre- to post-intervention was greater in the breathwork than in the meditation group (Figure 5). Pairwise comparisons confirmed a significant between-group difference in change, *p* = 0.007. Median PROMIS scores dropped from 54.8 to 39.2 in the breathwork group but remained relatively stable in the meditation group. A significant correlation was found between sleep-related impairment and total mystical experience (ρ = −0.62, *p* = 0.001, Bonferroni-adjusted *p* = 0.007), along with oneness/mystical (ρ = −0.62, *p* = 0.001, adjusted *p* = 0.006). Additionally, there was a significant correlation with oceanic boundlessness (ρ = −0.58, *p* = 0.003, adjusted *p* = 0.014).

#### Fatigue

Non-parametric factorial analysis revealed a significant main effect of group, *F*(1, 22) = 6.28, *p* = 0.020, indicating that SFQ scores were higher in breathwork relative to meditation participants overall (Figure 5). However, there was no significant main effect of time, *p* = 0.215, and no group × time interaction, *p* = 0.159, suggesting that the change in fatigue over time did not differ significantly between the two groups. Descriptively, fatigue levels actually increased in the breathwork group from a median of 16.5 to 19.0, whereas the meditation group remained relatively stable. There were no significant correlations between the primary outcomes and changes in fatigue.

#### Breathwork general experience

In the breathwork group, 11 of 12 participants responded to the question on their experience during the session and/or overall study period. No participants reported adverse events. All of the statements were positive in sentiment. Four participants described the breathwork as very profound, great/incredible, psychedelic in nature and relaxing. Another participant’s experience was focused on their body, whilst someone mentioned feeling most benefits and changes during and immediately after the session, subsiding after a few days. One mentioned the setting in particular:

*“I felt very supported and safe to discuss my experience and the community component was really beneficial as much as the experience itself.”*

Four participants in particular described experiencing particularly strong emotional releases:

“*I had a huge emotional release during the session, all explainable but what was interesting was [that] I heard my own voice saying to me – and the way I had previously interpreted it – “it’s OK.” It wasn’t in a sympathetic tone of it’s all [going to] be OK style, it was the understanding that clearly came with it, that it’s OK to cry, and much needed. Even though I see myself as someone who wells up quite easily, it’s not usually about/for myself: which I then realized I battle with [this]! A good insight and shift happened*… *and I’ve slept so much better all week even through most of the full moon and felt generally better in myself and capability to manage stress.*”

“*Very interesting visual hallucinations. Very cathartic emotional release. Very relaxing experience*.”

“*This experience of breathwork was more intense physically than any other time, and I was surprised to also experience more psychedelic qualities than I had experienced before. The experience was emotional and cathartic, at one time I cried but not with sadness, more with a strong feeling of what life is all about and how incredible it is and how lucky we are. Difficult solutions with people and problems felt easily resolvable by being loving, authentic and pure.*”

“*The session was excellently guided and very enjoyable. I didn’t have an experience that I’d categorize as psychedelic in any way though – I was fully present in the space throughout and had a predominantly physical experience of release. At times I felt emotional and, as the session progressed, I did feel more relaxed, within a flow, connected to the ground, to the people closest to me (past and present) and enormously grateful for my flawed life as a whole, as well as the people I was sharing the room with. [*…*] Thank you*… *to the guides, they were excellent and inspirational.*”

#### Meditation general experience

Seven of the 12 participants from the meditation group provided comments on their experience during the session and/or overall study period. No participants reported adverse events. All of the statements appeared positive in sentiment. One participant mentioned a small but recognizable change in non-judgmental awareness. Two stated that they really enjoyed the setting of the meditation (one found it deeply relaxing and restful), with one mentioning that it made it a particularly lovely, positive and relaxing experience. Another mentioned:

“*There was no profound insight gathered during the meditation session. I meditate daily, and my meditations are a bit more advanced rather than a body scan. However, it was good to go back to basics and focus on being in the present moment. Also to work through my feelings of not being in the breathwork session.*”

One participant mentioned:

“*I enjoy Yoga Nidra and meditation*… *I use these for relaxation mostly and restoration, not typically for deeper work to invite insight and thought/behavior shifts*.”

Lastly, another participant touched on some behavior shift paired with emotional relief:

“*Doing the meditation*… *I think had a positive influence on me as I have been able to meditate daily for the last week, and I have not been able to do that for a few months. There are always many factors of healing I do, so I can’t fully attribute it to this, but I think it helped. I felt terrible the morning and few days before [the session]*… *and the meditation was a little break from that. Coming back to wakefulness after the meditation was hard, but it was good to get some relief from the mental/soul pain during the body scan*.”

## Discussion

To the best of our knowledge, we present the first attempt at a controlled study to date on breathwork and ASCs. In this exploratory investigation, participants engaging in a single session of HVB, specifically CCB, were associated with reporting more profound ASCs compared to those practicing body scan meditation. The breathwork group demonstrated significantly greater mystical experiences overall compared to the meditation group, with large effect sizes observed across multiple dimensions. In particular, breathwork participants reported higher scores for total mystical experience, oneness, positive mood and ineffability. These participants also experienced significantly greater emotional breakthrough compared to meditation participants, with a moderate effect size. This finding is consistent with the alleged “cathartic” nature of breathwork.

Further, breathwork was associated with significantly greater ASCs regarding oceanic boundlessness and visionary restructuralisation, both showing large effect sizes. We were not able to detect significant correlations between scores on the IPPS and any of the ASC primary outcome measures, likely due to insufficient statistical power. A much larger sample size would be required to confirm such an observation, given the possibilities of high variability in participants’ predicted response to psychedelic experiences, and its possibly weak association to their actual responses to breathwork or meditation.

Overall we found similar results to Bahi et al. (2024) and Havenith et al. (2025) with regards to psychedelic-like effects of breathwork. These two HVB studies broke down the core components of the 5D-ASC into its further 11 subscales (Studerus et al., 2010) and compared their breathwork data with that of psychedelic compounds as per the Altered States Database (Prugger et al., 2022).

Accordingly, we did the same and compared our results against both Havenith et al. (2025) (see Supplementary Figure 2) and Bahi et al. (2024) (Supplementary Figure 3). Owing to the exploratory nature of this, quantitative analysis was not conducted. Notwithstanding its limitations, visual inspection supported results of our primary outcome analysis, highlighting a large impact of breathwork on oceanic boundlessness and its component parts/subscales (insightfulness, spiritual experience, experience of unity, blissful state, disembodiment). In fact, our breathwork intervention was associated with higher levels of oceanic boundlessness. The reason for the potentially more powerful and prominent impact of the present breathwork could be due to the more ecologically valid setting compared to Bahi et al. (2024) (delivered in individual session formats only thereby potentially removing key elements pertaining to set and setting) without repeated interruptions throughout to measure intensity of the experience as in Havenith et al. (2025).

Amongst the secondary outcomes, the breathwork group self-reported substantially greater psychological insight and accompanied behavioral change (measured by the PIS) compared to the meditation group, with large effect sizes observed for both measures. This finding suggests that breathwork could be connected to meaningful psychological breakthroughs, accompanied by translation of these insights into tangible behavioral modifications. A significant group × time interaction revealed that breathwork participants reported greater improvements in sleep-related impairment than meditation participants, with median scores decreasing markedly in the breathwork group whilst remaining stable in the meditation group. These changes were significantly correlated with total mystical experience, oneness, and oceanic boundlessness, suggesting that the depth of ASCs may have been related to sleep benefits. Sleeping is a common experience which, in itself, creates ASCs (Srinivasan, 2015). Importantly, sleep facilitates insight, a mental reorganization that results in a sudden acquisition of explicit knowledge, enabling a qualitatively different form of behavior (Wagner et al., 2004), and is essential for consolidating various types of memory, supporting insightful and inferential thinking (Deak and Stickgold, 2010).

Both groups demonstrated significant pre-post-intervention reductions in stress, anxiety, and depression. No significant between-group differences in change were detected. However, participants in the meditation group presented with higher baseline DASS depression and anxiety scores and exhibited numerically greater absolute reductions. Within the meditation group, baseline depression severity was strongly negatively associated with magnitude of change (ρ = −0.75, *p* = 0.005), indicating that individuals with higher initial symptom levels experienced larger reductions. In contrast, the HVB group exhibited very low baseline depression scores, which may have constrained observable improvement and reduced power to detect antidepressant superiority, consistent with a potential floor effect and baseline-dependent change. Similarly, both groups demonstrated significant improvements in mental wellbeing over time, suggesting that both breathwork and meditation effectively enhanced psychological wellness to a similar degree.

Because statistical power was constrained by the sample size, only relatively large effects could be detected with confidence. Thus, our findings should be interpreted in light of its limited statistical power. While a few analyses produced effect sizes of meaningful magnitude, these did not reach statistical significance, suggesting a high probability that smaller but potentially relevant effects were not detectable within the current sample. For example, numerical between-group differences for transcendence (of time and space), auditory alterations, vigilance reduction, dread of ego dissolution, or depersonalization were not statistically significant. Further, there was no statistically significant difference between practices with regard to effects on psychological wellbeing scores over the observed timeframe. Finally, no significant changes in fatigue were observed over time in either group.

No participants reported adverse events, however this is unsurprising as they were recruited on the basis of having practise CCB before without experiencing such phenomena. We deemed it more ethically sound to conduct a first attempt at an experimental study of this nature on CCB and ASCs in individuals with some experience due to physical safety and the possibility of adverse effects from HVB.

Participants in the breathwork group reported transformative experiences. The very limited open-text qualitative data we collected revealed that some participants described their sessions using terms such as profound, incredible, and psychedelic, indicating the depth of subjective experience achieved through the breathwork session. A particularly salient theme was the occurrence of significant emotional releases, with a third of breathwork participants specifically describing cathartic experiences during their sessions.

Qualitatively and complementary, though not confirmatory, in line with significant quantitative results relating to psychological insight and behavior change as per the PIS, the emotional processing facilitated by breathwork appeared to extend beyond the immediate session, with participants reporting insights into their emotional patterns and improved stress management capabilities. One participant noted enhanced sleep quality and stress resilience lasting throughout the week following the intervention. There were psychedelic-like qualities reported by some participants, including visual hallucinations and ASCs. The group setting also emerged as a crucial factor, with participants highlighting the importance of feeling safe and supported during the session. This underscores the significance of appropriate set and setting in breathwork interventions, with priming and integration potentially being just as, if not more, imperative than the respiratory component which may be simply acting as a catalytic stimulus for change (Fincham et al., 2024).

The meditation group qualitatively, again though not confirmatory, reported more subtle but still meaningful changes. Participants reported increased non-judgmental awareness and found the structured setting particularly beneficial for relaxation and restoration. Notably, one participant experienced renewed motivation for daily meditation practice following the intervention, suggesting potential behavioral spillover effects. The qualitative feedback (again, very limited) indicated that whilst the meditation intervention was less dramatic than breathwork, it provided valuable grounding and present-moment awareness. Some participants noted that the body scan meditation offered relief from psychological distress, though the changes were generally described as less profound than those experienced in the breathwork group. While the sentiment of the qualitative data was positive in both groups, this other outcome was simply intended to complement the primary and secondary outcomes.

In summary, this exploratory study found that breathwork was associated with larger acute psychedelic-like experiences than meditation, an active comparator chosen to control for set and setting, alongside greater emotional breakthrough and mystical experience. Sub-acute associations were also observed, with breathwork participants reporting substantially greater psychological insight and behavioral change 1 week later. Both groups showed comparable improvement in most health and wellbeing outcomes over time, though a group × time interaction suggested that sleep-related impairment may have benefited more from breathwork than meditation following a single session. Taken together, these preliminary observations provide initial support for the feasibility and acceptability of ASC-focused breathwork research and generate potential hypotheses for future confirmatory trials in clinical populations.

A recent systematic review and meta-analysis confirmed that the intensity of the psychedelic experience is reliably associated with clinical improvements (Romeo et al., 2025), although it focused exclusively on psychedelic compounds. Interestingly, Havenith et al. (2025) found a significant correlation between intensity of ASCs experienced during breathwork and changes in wellbeing (though not with depressive symptoms), suggesting that the intensity of ASCs might mediate some therapeutic effects of breathwork. Our results extend Havenith et al.’s (2025) findings to a degree, further supporting the hypothesis that CCB has promising clinical therapeutic potential mediated by induction of powerful ASCs. However, this hypothesis will require involving participants with no prior experience of breathwork, and controlled studies in clinical populations with raised baseline levels of psychological difficulties, to assess tolerability and signals of efficacy.

## Limitations and future directions for research

Several limitations warrant consideration when interpreting these findings. For example, though not statistically significant, baseline depression scores appeared substantially different between groups. This could have limited the capacity to detect antidepressant effects (floor effect), and may have inflated apparent improvements in the meditation group. A detailed qualitative framework, with in-depth interviews evaluated through appropriate coding strategies and thematic analysis, would also benefit and enrich future research. Moreover, there was not opportunity to have a true pre-test assessment conducted for the primary ASC state outcome measures, making it difficult to interpret changes as intervention “effects” or improvements, especially within a single-session design. Additionally, the 7–10 day assessment gap for secondary trait outcomes may have introduced substantial influence from unrelated psychological, environmental, or lifestyle factors affecting wellbeing outcomes independent of the intervention. Given these limitations, the study may be better interpreted as a preliminary phenomenological exploration rather than an efficacy-oriented investigation.

The open-label study design, modest sample size, limited follow-up periods, and absence of physiological parameters to confirm differential breathing patterns in the CCB group represent key methodological constraints. Whilst assessment of physiological parameters was beyond the scope of this study, future investigations with greater study lengths and larger samples could potentially elicit between-group differences over time. Our design choices prioritized the ecological validity of setting and context, which are known to influence the intensity of breathwork-induced ASCs as reported by Kartar et al. (2025).

The CCB and comparator conditions were not identical and differed beyond the core ventilatory rate manipulation. We deemed the body scan an apt comparator because it allowed us to account for set and setting – one of the most difficult aspects to control in this research – and was not entirely opposed to ASC-induction, as prior research has shown mindfulness to be associated with such experiences (Galante et al., 2024). However, the CCB session was longer and incorporated intense musical accompaniment, known to influence emotional processing and psychological insight. The duration of body scan meditation approximated that of similar practices taught in MBSR and was not extended to prevent participants falling asleep. Future studies could implement a longer sham preparation and/or integration session. We selected soft background music from portions of the CCB playlist to maintain ecological validity, matching the lower intensity, relaxing aspects of the CCB session to the body scan meditation.

Critically, the CCB condition involved explicit priming for psychedelic-like experiences (facilitators discussed potentially expanded consciousness states and prepared participants for phenomena including body temperature fluctuation, tetany and emotional expression) whereas the comparator lacked such framing. Whilst we chose this approach to ensure ecological validity, this may have introduced substantial expectancy effects as our sample comprised individuals with prior breathwork experience, and therefore with potential pre-existing beliefs about its psychedelic-like qualities which could have influenced their subjective experiences and ratings. Research demonstrates that hypnotic suggestion alone can produce mystical-type experiences in laboratory settings (Lynn and Evans, 2017), suggesting our findings may partially reflect expectancy effects rather than purely physiological consequences of hyperventilation.

A notable limitation of the present study is the absence of a pre-session expectancy measure. Given that the IPPS includes items assessing intentionality (which may partially capture expectancy) we cannot rule out that subjective ASC intensity was influenced by participant expectations. Future studies should incorporate validated expectancy scales administered prior to the session to enable covariate adjustment and examine expectancy as a potential moderator of ASC depth.

Expectancy effects might complicate generalizability, particularly given the inclusion of couples. However, sensitivity analysis excluding dyads showed that only the MEQ variable of oneness/mystical lost significance when corrected for (*p* = 0.09), whilst retaining a large effect size (*r* = 0.56). It is important to note that we used conservative correction approaches in a very small sample. Whilst we assessed basic demographic characteristics, we did not systematically collect data on exact amount of breathwork practice, meditation experience, or recruitment contexts (e.g., specific breathwork organizations or communities). Such information would have been valuable for exploring whether breathwork/meditation expertise, which itself may produce ASCs, partially explained the observed outcomes.

These considerations are crucial to inform and refine future designs of studies investigating the effects of CCB practices. First, the preparatory elements and intense music are ecologically valid components of CCB practice; omitting warnings about tetany and other pronounced physiological reactions would be ethically problematic, whilst substituting CCB’s characteristically intense soundscapes with gentle meditation music would fundamentally alter the intervention. Future research might explore dismantling designs to parse the relative contributions of ventilatory patterns, musical accompaniment, session duration, and preparatory framing.

A logical next step would be replicating our study in breathwork-naïve participants to help mitigate expectancy effects and determine whether such ASCs emerge as profoundly as in experienced practitioners. Future research contrasting HVB effects across participants with varying experience levels and in different environments is essential for informing the public about safe breathwork engagement. Such (breath)work can guide careful and personalized selection of appropriate techniques, settings and environments, thereby minimizing potential adverse effects (Fincham et al., 2023a).

More generally, a significant limitation in the current literature is the tendency to treat altered states as a unitary outcome, representing a conceptually weak approach. ASCs are frequently discussed as though they form a single dimension varying only in intensity [there are now even attempts to create non-hallucinogenic psychedelics to reduce intense, acute ASCs which may limit scalability (Chen et al., 2025)]. However, psychedelic states, breathwork-induced hyperventilation states, meditative absorptions (e.g., *jhāna*), and non-dual awareness are phenomenologically and mechanistically distinct phenomena, despite exhibiting superficial overlap on standardized questionnaire measures. To advance the scientific study of ASCs, it is essential to pair empirical claims with detailed phenomenological accounts and strong conceptual foundations, ensuring a clear and comprehensive map of these complex states.

## Data Availability

The datasets presented in this study can be found in online repositories. The names of the repository/repositories and accession number(s) can be found in the article/Supplementary material.

## Footnotes

1
https://www.qualtrics.com/

2
https://www.stacihaines.com/

3
https://www.healthmeasures.net/

4
https://warwick.ac.uk/
