# Supplementary material for: An exploratory study of breathwork-induced altered states of consciousness in experienced practitioners: the airways to alteration (A2A) trial
Source: Front Psychol. 2026 Jun 10;17:1851882. doi: 10.3389/fpsyg.2026.1851882 (PMC13291248; doi:10.3389/fpsyg.2026.1851882)

Supplementary material

An exploratory study of breathwork-induced altered states of consciousness in experienced practitioners: the Airways to Alteration (A2A) trial

Guy W. Fincham[1*], Edward Caddye[1], Amy A. Kartar[1], Elizabeth A. Lilley[2], Nicola Stoke[3], Alessandro Colasanti[1,4]

[1] Breathwork Lab, Department of Clinical Neuroscience, Brighton & Sussex Medical School, UK
[2] Psychoactive Trials Group, Institute of Psychiatry, Psychology & Neuroscience, Kings College London, UK
[3] Independent Scholar, UK
[4] Sussex Partnership NHS Foundation Trust, UK
* Corresponding author

Supplementary Figure 1. Study participant flow.

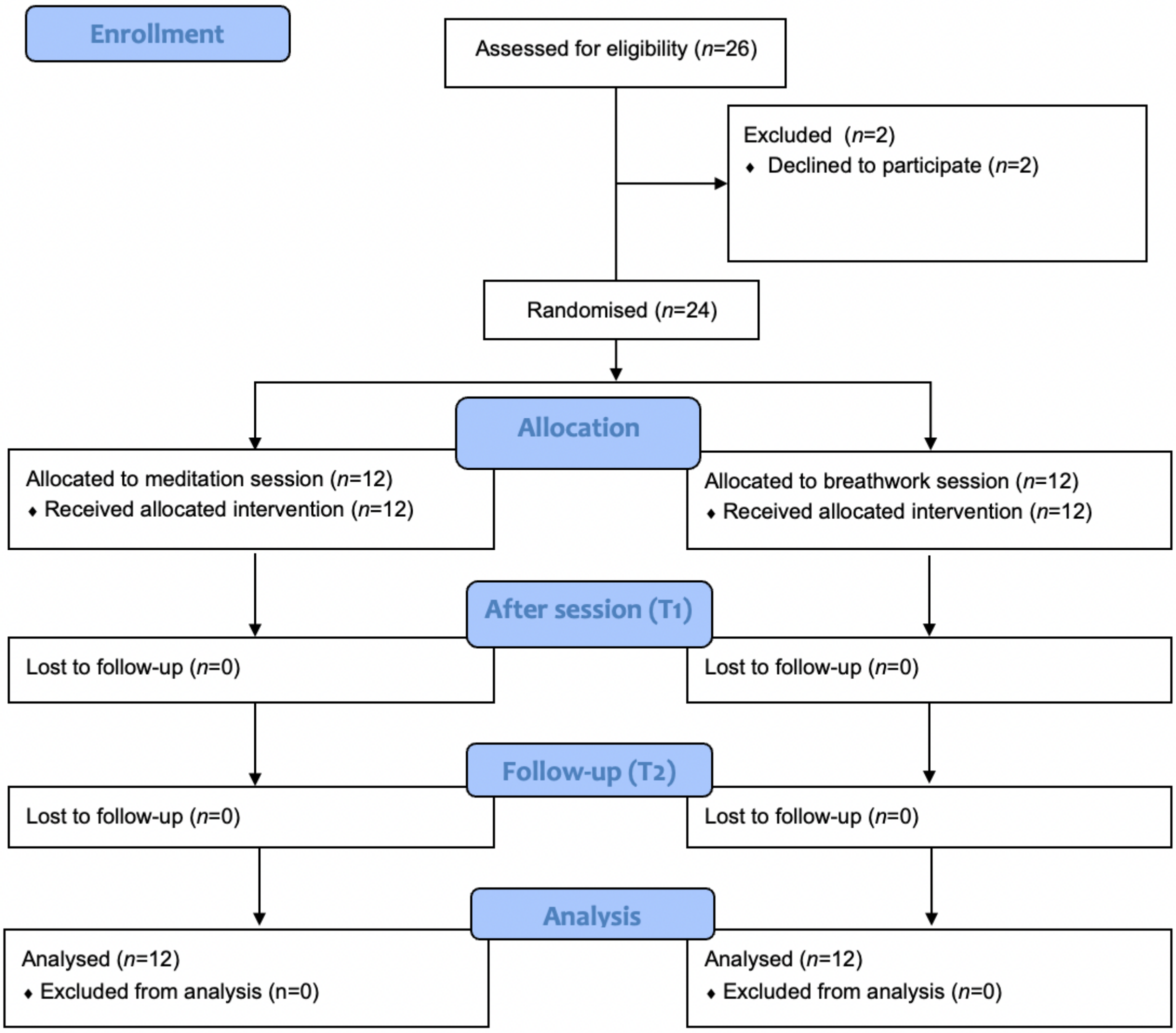

Anonymised data included in this study are available on the repository Zenodo: 10.5281/zenodo.16410513.

Supplementary Figure 2. Mean 11-Dimensions of Altered States of Consciousness Scale scores by intervention, with scores averaged across participants for each subscale. Adapted from Havenith et al. (2025) using their breathwork interventions (90-min and 180-min), along with data extracted by the authors from the Altered States Database: Psilocybin oral (scores pooled across five relevant clinical studies), LSD oral/intravenous (pooled across six studies) and 0.125 mg MDMA oral (five studies), along with placebo treatments (three clinical studies). Green and orange thick solid lines highlight our current study's 90-min breathwork and 40-min meditation groups, respectively.

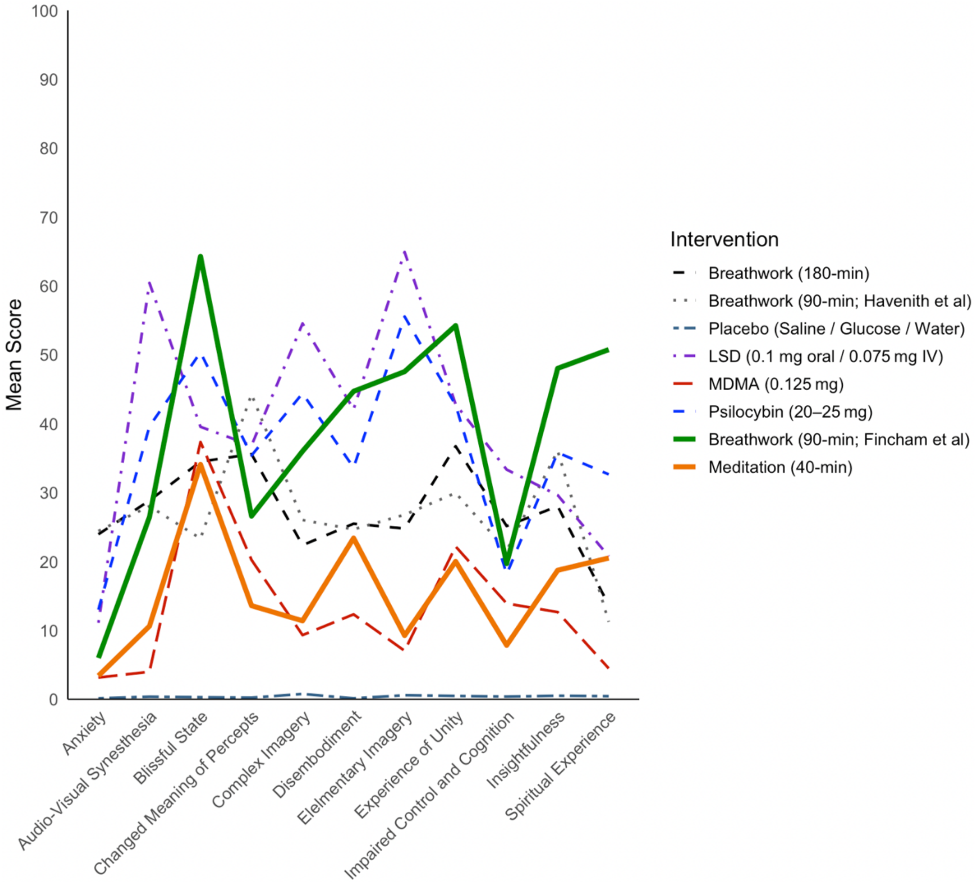

Supplementary Figure 3. Phenomenological profiles of breathwork and meditation compared to dose-dependent phenomenological profiles of psilocybin on 11-Dimensions of Altered States of Consciousness Scale mean scores. Adapted from Bahi et al. (2024) using data extracted by the authors from the Altered States Database, along with their 45-min breathwork intervention. Data from our current study are represented by green (90-min breathwork) and orange (40-min meditation) thick solid lines.

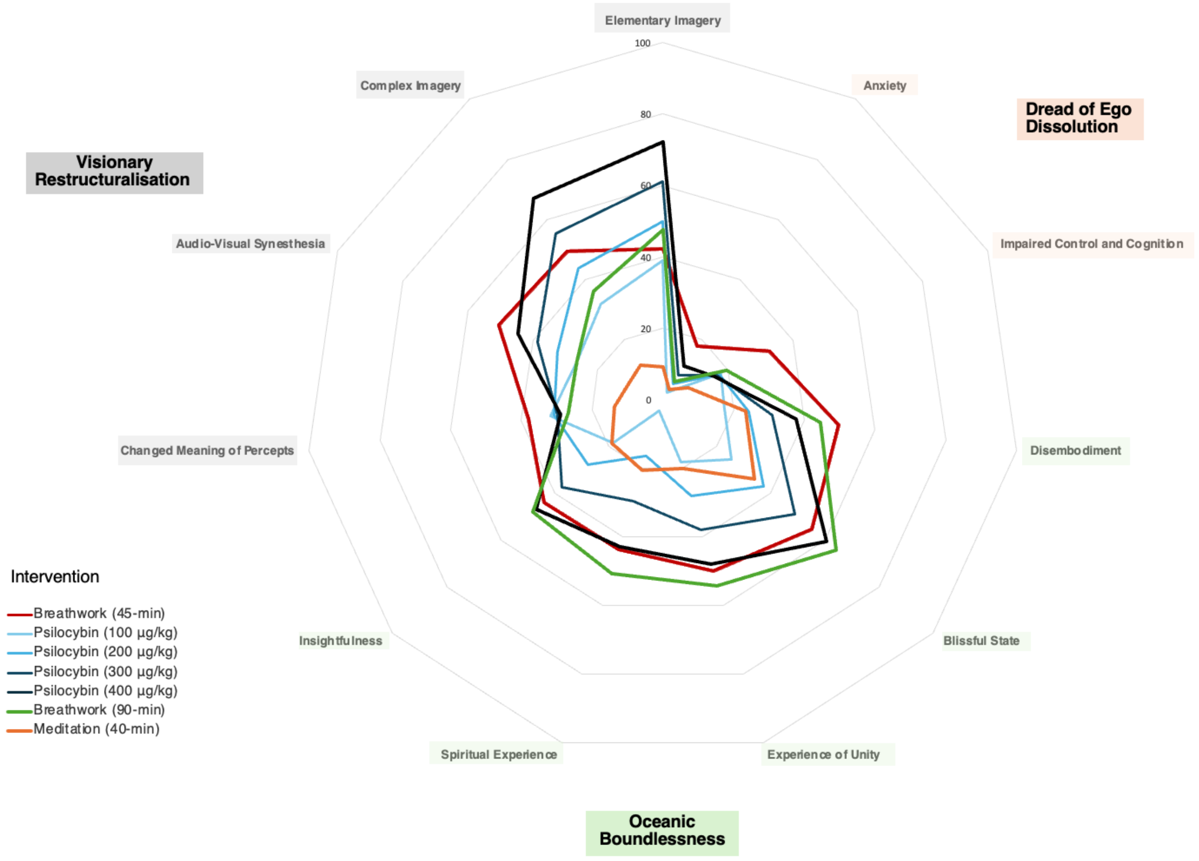

Supplement: Supplementary file 1 [file Supplementary_file_1.pdf]
